# Textual Features and Risk Preference Effects on Mental Health Education Among Teenager Students in Chongqing, China

**DOI:** 10.3389/fpsyg.2022.911955

**Published:** 2022-05-26

**Authors:** Mengyao Jiang, Zuyue Zhang, Li Kang, Jing Liao, Shumin Wang, Yalan Lv, Xiaoyu Zhou, Xiaorong Hou

**Affiliations:** ^1^College of Medical Informatics, Chongqing Medical University, Chongqing, China; ^2^Medical Data Science Academy, Chongqing Medical University, Chongqing, China; ^3^Chongqing Engineering Research Center for Clinical Big Data and Drug Evaluation, Chongqing, China; ^4^Chongqing Tianjiabing Middle School, Chongqing, China

**Keywords:** mental health, textual features, risk preference, emotional polarity, teenager students

## Abstract

**Background:**

Mental health is a public health problem of great concern. Previous studies show that textual features and individual psychological characteristics can influence the effect of receiving information.

**Purpose:**

This study explores whether textual features influence the persuasiveness of teenager students’ mental health education while considering the influence of risk preference.

**Methods:**

From November to December 2021, a cross-sectional study was conducted among 1,869 teenager students in grade 7–12 in Chongqing, China. Wilcoxon signed-rank test, multiple logistic regression, and subgroup analysis were used to analyze the data.

**Results:**

Among the four textual features mentioned in this study, a significant difference was reported in the persuasive effects of information with and without numerical features (*p* < 0.001), and such information tended to include digital features. The result for the symbolic features (*p* < 0.001) was consistent with the numerical features. The persuasive effects of positive and negative emotional information significantly differed (*p* < 0.001), with the former showing a better performance. No significant differences were observed between the persuasive effects of information with and without emotional conflicts (*p* > 0.05). Combined with those from the risk preference analysis, results showed that the regulatory effect of risk preference was only reflected in emotional conflicts. Students who prefer having no emotional conflict in the text showed the characteristics of risk avoidance, or lower grades, or rural or school accommodation. Most teenager students are also risk averse, especially females (or = 2.223, 95%CI:1.755–2.815) and juniors (or = 1.533, 95%CI: 1.198–1.963).

**Conclusion:**

The numbers, symbols, and positive emotions in the text generate an active effect on teenager students receiving mental health education. Students avoiding risk are inclined to read texts without emotional conflicts. The probability of male choosing texts with positive emotional polarity is 33.5% lower than that of female. Female students and those from lower grades also demonstrate a higher inclination to risk avoidance compared with their male and higher grade counterparts. Therefore, educational materials with different text characteristics should be developed for teenager students with varying characteristics.

## Introduction

The Adolescent Mental Health (2021) report published by WHO proposed that depression, anxiety, and behavioral disorders are the leading causes of illnesses and suicides among adolescents. Globally, 1 in 7 individuals aged between 10- and 19-year experience a mental disorder, accounting for 13% of people belonging to this age group. Suicide is the fourth leading cause of death among people aged 15 to 19 years. In addition, the China National Mental Health Development Report (2019–2020) recorded that at least 24.6% of adolescents in China are facing depression in 2020, with those reporting severe depression accounting for 7.4%. Many factors can affect the wellbeing and mental health of adolescents, including violence, poverty, stigma, exclusion, and living in humanitarian and fragile settings (Man et al., 2022). The consequences of not addressing these mental health conditions may extend to adulthood and impair both the physical and mental health of adults and limiting their access to opportunities to lead fulfilling lives (Reef et al., 2011; Davis et al., 2021). Mental health education is an effective strategy to prevent psychological problems. In particular, schools provide psychosocial support for teenagers to improve their mental health (Pannebakker et al., 2019). The degree of message receiving is an effective indicator for evaluating the effect of health education, and information acceptance is directly affected by texts (Ghose and Ipeirotis, 2011; Thakkar et al., 2016).

Many studies have shown that information containing textual features can affect the choices of individuals in different situations. For instance, E. Joyce et al. found that text with positive emotions can enhance the community identity of individuals and encourage their participation (Joyce and Kraut, 2006). Szuchewycz et al. found that text with clear language, firm tone, emotional color, and specific rhetoric is highly persuasive (Szuchewycz, 1995). Numerous studies have also shown that the length of the text, the placement of certain words and symbols, and the use of numbers increase the impact of a text (Jiang et al., 2019; Pengnate, 2019). Why do texts with distinct features have different effects even when they express the same idea? In health, the text is considered the most basic means of intervention. In mental health intervention, information with different characteristics may easily trigger sensitive responses from an audience. Although many studies have highlighted the effect of textual features on information persuasion, only few have systematically investigated such effect on the mental health of specific groups.

Besides the information itself, the characteristics of the audience also affect the persuasiveness of a text. The preferences of adolescents for EQ-5D-Y-3 l health states differ from those of adults and children (Prevolnik Rupel et al., 2021). The differences in the adolescent and adult values for identical health states are highly profound and may be significant enough to affect health care policies (Ratcliffe et al., 2012). From the perspective of natural language processing, some scholars believe that age can result in reading differences, whereas others believe that males and females have different understandings of the same text. These studies show that the characteristics of the population have a certain impact on the reception effect of information (Cheng et al., 2011; Shake et al., 2016; Zhu et al., 2018). Therefore, the forms and methods of education need to be studied based on the characteristics of the population.

In addition to basic population characteristics (e.g., gender and age), psychological characteristics also warrant attention. Risk preference is a relatively stable and identifiable psychological trait (William and Gartner, 2017), which could be affected by risk perception and consistent with risk tendency (Sitkin and Pablo, 1992). As one of the most important influencing factors in psychological decision-making, risk preference has always been the emphasis of behavioral decision-making research. Previous studies show that the information type affects the risk perception of the audience (Perko, 2014). Risk perceptions may have deliberative, affective, and experiential components, with the impact of emotion on risk perception and risk preference being the most obvious. While positive situations encourage conservative attitudes, negative situations drive individuals to engage in risky decision-making (Taber et al., 2015; Li-jing et al., 2017). Moreover, those interventions that change the risk perceptions of individuals can also change their health behaviors (Ferrer and Klein, 2015). Other studies have shown that individual risk preferences and attitudes affect their health behaviors (Qiu, 2011).

In view of the above arguments, this study explores some strategies for improving the effect of mental health education on teenager students in grade 7–12 while taking the risk preferences of these students and the characteristics of texts into account.

## Materials and Methods

### Study Design

A cross-sectional study on the persuasive effects of textual features and crowd characteristics on the psychological health education of teenager students in grade 7–12 was conducted in Chongqing, China from October to December 2021. A self-administered paper questionnaire was administered among participants who were selected *via* convenient sampling. The persuasive effect of textual features and other characteristics, including risk preference, gender, age, place of residence, and boarding, on the mental health education of teenager students in grade 7–12 was explored. The interaction among these crowd characteristics was also investigated.

### Participant Selection

The participants should be teenager students in grade 7–12 given that they are in an important period of mental health education that may affect their psychological and physiological states.

### Questionnaire

This questionnaire was developed based on Article 9 of the existing Core Information and Interpretation of Chinese Adolescent Health Education (2018 Edition). The questionnaire contents were finalized after several discussions by an expert group. Our expert group contains a statistician, a psychologist, and a public health expert. The questionnaire was divided into three parts, namely, essential information (including demographic characteristics), experience materials with textual features, and a risk preference scale.

PART I: Following previous studies (Mcgee et al., 2014; Liu and Niu, 2016; Zhu et al., 2018) this part of the questionnaire included questions related to the demographic characteristics of the participants, including their gender, age, grade, type of school (i.e., boarding school or not), and place of residence (i.e., rural or urban).

PART II: The experience materials with textual features were designed as follows. First, four textual features were identified from previous research (unpublished data) *via* a literature review (Pengnate, 2019; Guo Liu, 2020), namely, emotional conflict, numerical features, symbolic features, and emotional polarity. Afterward, the theme “mental health education during an epidemic” was identified (King, 2021). As shown in Table 1, each of the selected features has two opposing options, namely, with and without textual features. A five-point Likert scale was designed, and the average and median of the participants’ responses were used to measure the persuasion effect of a certain type of information.

**Table 1:** Experience materials with textual features.

Theme	Option	
	With textual features	Without textual features
Emotional conflict	From “Social phobia” to “Social Whiz”—a step-by-step guide to social nerds (5 4 3 2 1)^a^	Correctly deal with the change of social interaction and learn how to become a social talent (5 4 3 2 1)^a^
Numerical features	3 tips help you to say goodbye to all anxiety (5 4 3 2 1)^a^	Help you to say goodbye to all anxiety (5 4 3 2 1)^a^
Symbolic features	Anxiety (• _ •)? Panic!? Teach you how to prevent the epidemic, refuse to panic!! (5 4 3 2 1)^a^	Anxiety, panic, teach you how to prevent the epidemic, refuse to panic (5 4 3 2 1)^a^
Emotional polarity	Being with us, the world is bright (5 4 3 2 1)^a^	Isolated, the world becomes anxious (5 4 3 2 1)^a^

^a^(5 = totally like, 4 = like, 3 = neutral, 2 = dislike, and 1 = totally dislike).

PART III: The risk preference scale was designed following Hsee and Weber (1997), and Weber (1999) and is shown in Table 2.

**Table 2:** Risk preference scale.

Set	Question	Sure option	Risky option
1	1	Receive $400 for sure	Flip a coin; receive $2000 if H^a^or $0 if T^b^
1	2	Receive $600 for sure	Flip a coin; receive $2000 if H^a^ or $0 if T^b^
1	3	Receive $800 for sure	Flip a coin; receive $2000 if H^a^ or $0 if T^b^
1	4	Receive $1,000 for sure	Flip a coin; receive $2000 if Ha or $0 if T^b^
1	5	Receive $1,200 for sure	Flip a coin; receive $2000 if H^a^ or $0 if T^b^
1	6	Receive $1,400 for sure	Flip a coin; receive $2000 if H^a^ or $0 if T^b^
1	7	Receive $1,600 for sure	Flip a coin; receive $2000 if H^a^ or $0 if T^b^
2	1	Receive $20 for sure	Flip a coin; receive $100 if H^a^ or $0 if T^b^
2	2	Receive $30 for sure	Flip a coin; receive $100 if H^a^ or $0 if T^b^
2	3	Receive $40 for sure	Flip a coin; receive $100 if H^a^ or $0 if T^b^
2	4	receive $50 for sure	flip a coin; receive $100 if H^a^ or $0 if T^b^
2	5	Receive $60 for sure	Flip a coin; receive $100 if H^a^ or $0 if T^b^
2	6	Receive $70 for sure	Flip a coin; receive $100 if H^a^ or $0 if T^b^
2	7	Receive $80 for sure	Flip a coin; receive $100 if H^a^ or $0 if T^b^

^a^H means the coin head shows up; ^b^T means the coin tail shows up.

In scenario 1, the participants were asked to imagine that they bought a lottery ticket a week ago. They were then informed that they have won the lottery and have two options to get the money. The participants then read two sets of questions, with the first set containing large outcome size questions and the second set containing small outcome size questions. Each set included seven questions. The options for these questions are listed in Table 2.

These questions were printed in a random order, except that the first set always preceded the second set. In scenario 2, the questions were preceded by the following instructions:

Suppose that you violated a traffic rule and hurt somebody a week ago. You are now informed that you will be fined and have been given two options on how to pay the fine.

The participants then answered made the same two sets of questions listed above, except the word “receive” was replaced by “pay.”

In November 2021, a pre-survey was conducted among 55 teenager students to test the reliability of the questionnaire. Cronbach’s alpha of the questionnaire was 0.765.

### Data Collection

After answering demographic questions, the participants were given experience materials with textual features that contained eight options, with each option rated on a scale of 1 to 5, indicating “totally dislike,” “dislike,” “neutral,” “like,” and “totally like,” respectively. The questionnaire results were the manually coded in a computer by the interviewers.

### Ethical Aspects

The study protocol was approved by the Ethics Committee of the Chongqing Medical University (record number 2018011). The participants gave their informed consent before answering the questionnaire.

### Statistical Analysis

The collected data were processed using Microsoft Excel before they were inputted into a database. Data analysis was performed using SPSS 25.0 (IBM Corporation, Armonk, NY, US). For the demographic characteristics of the participants, the frequencies and percentages were calculated for the categorical variables. According to the standards for calculating the risk preference index (Weber, 1999), the risk preference values recorded in the third part of the questionnaire were counted and grouped. A Wilcoxon signed-rank test was performed to check for a significant difference exists among different types of messages, and the computed median was used to represent the persuasion effect of a particular type of information. Multinomial logistic regression analysis was performed to investigate the factors that can affect the risk preference of the participants. The independent variables included gender, age, place of residence, and boarding. Subgroup analysis was performed to supplement the impact of participant characteristics (e.g., demographic statistical characteristics and population risk preference) on their text feature preference. A value of p of no greater than 0.05 was considered statistically significant.

### Quality Control

The questionnaire was modified several times after expert interviews and a pilot survey. The offline research team members, including three students (two postgraduate and one undergraduate), received a standardized investigation training. These investigators should understand the purpose and methodology of the study in detail and have extensive experience in dealing with potentially sensitive issues.

A total of 1,869 participants were recruited from 4 schools in Chongqing, China for an offline survey. After applying the exclusion criterion I, a total of 1,655 questionnaires were retained, which corresponded to an effective rate of 88.55%. After combining exclusion criteria I and II, a total of 1,469 questionnaires were retained, which corresponded to an effective rate of 78.59%. Cronbach’s alpha of these questionnaires was 0.729.

Exclusion criterion I: In the part of mental health materials with textual features, all the options of participants are consistent or missed.

Exclusion criterion II: According to the risk preference index (RP index) questionnaire and its evaluation method (Hsee and Weber, 1997), the questionnaire was deemed invalid if the responses of the participants were illogical (e.g., the positive benefit option is selected in question 2, whereas the risk option is selected in question 3).

## Results

### Demographic Characteristics of Participants

The demographic characteristics of the participants are presented in Table 3. The number of male and female participants was nearly the same (with males comprising 56.8% of the sample). Most of the participants were senior grade students (60.2%), the vast majority (85.8%) were living in urban, and 63.1% were boarders.

**Table 3:** Demographic characteristics of the participants (*n* = 1,469).

Variables	Group	Frequency (n) or sample percentage (%)
Gender	Female	834 (56.8%)
	Male	635 (43.2%)
Grade	Grade 7–9	584 (39.8%)
	Grade 10–12	885 (60.2%)
Place of residence	Urban	1,261 (85.8%)
	Rural	208 (14.2%)
Boarding mode	Board at school	927 (63.1%)
	Day school	542 (36.9%)

### Choices of Textual Features

Two options were experimentally manipulated, namely, with and without textual features. As shown in Table 4, the Wilcoxon signed-rank test was performed to analyze the differences in the persuasive effects of different options. Among the four textual features mentioned in this study, a significant difference was reported in the persuasion effects of the information with and without numerical features (*p* < 0.001), and such information tended to include digital features (larger median). The result for the symbolic feature (*p* < 0.001) was consistent with that of the numerical feature. The persuasion effects of positive and negative emotional information significantly differed (*p* < 0.001), with positive emotion showing a better effect (larger median). No significant differences were observed in the persuasion effects of information with and without emotional conflict (*p* > 0.05).

**Table 4:** Choices of textual features.

Options	Median (Average)	*Z*	*P*
**Emotional conflict**			
Have emotional conflict	3.55(3.49)	−1.958^a^	0.050
No emotional conflict	3.58(3.59)		
**Numerical features**			
Have numerical features	4.02(3.89)	−19.322^b^	0.000^**^
No numerical features	3.27(3.26)		
**Symbolic features**			
Have symbolic features	4.07(3.91)	−19.542^a^	0.000^**^
No symbolic features	3.24(3.23)		
**Emotional polarity**			
Positive	4.00(3.42)	−15.167^a^	0.000^**^
Negativity	3.90(3.39)		

^a^Based on negative rank; ^b^Based on positive rank; Wilcoxon signed-rank test was used, ^*^ and ^**^ denote statistical significance at *p* < 0.05 and *p* < 0.001, respectively.

### Influence of Risk Preference on Choice of Textual Features

A subgroup analysis was performed to determine whether the crowd characteristics of risk preference, gender, age, place of residence, and boarding affect the participants’ choice of textual features. Results in Table 5 show that risk preference only affects the text feature of emotional conflict. Students who prefer having no emotional conflict in the text showed the characteristics of risk avoidance, or lower grades, or rural or school accommodation (*p* < 0.05). At the same time, the risk preference and demographic factors were taken as independent variables, and the characteristic preference was taken as dependent variable. By using binary logistic regression analysis, it was found that when the characteristics of emotional conflict and emotional polarity worked as dependent variables, the regression equation could be established successfully. The results of Table 6 show that the older the age, the higher the probability of choosing texts with emotional conflict, which reflected as that the probability of choosing texts with emotional conflict characteristics is 1.126 times higher than the original for every 1-year-old increase in age; and when compared with the risk aversion type, the probability of choosing texts with emotional conflict characteristics is 1.086 times for risk-seeking type. In Table 7, the probability of men choosing texts with positive emotional polarity is 33.5% lower than that of women; Moreover, the probability of choosing positive emotional polarity reduced by 8% for every 1-year-old increase in age as well.

**Table 5:** Influence of risk preference on choice of textual features.

Variables	Group	*Z*	*P*
Gender	Female	−1.383^a^	0.167
	Male	−1.419^a^	0.156
Grade	Grade 7–9	−2.300^a^	0.021^*^
	Grade 10–12	−0.726^a^	0.468
Place of residence	Urban	−1.127^a^	0.26
	Rural	−2.245^a^	0.025^*^
Boarding mode	Board at school	−2.627^a^	0.009^*^
	Day school	−.296^a^	0.767
Risk preference	Risk avoidance	−2.190^a^	0.029^*^
	Risk-seeking	−0.422^a^	0.673

^a^Based on negative rank; Wilcoxon signed-rank test was used, ^*^ and ^**^ denote statistical significance at *p* < 0.05 and *p* < 0.001, respectively.

**Table 6:** Variables in the equation (Features of emotional conflict).

Variables		B	S.E.	Wald	df	Sig.	EXP(B)	95% C.I. for EXP(B)	
								Lower	Upper
Step 3^a^	Age	0.119	0.037	10.464	1	0.001^*^	1.126	1.048	1.611
	RPI	0.082	0.036	5.200	1	0.023^*^	1.086	1.102	1.645
	Constant	−2.833	0.571	24.572	1	0.000^**^	0.059		

^a^Variable(s) entered on step 3: sex; ^*^ and ^**^ indicate statistical significance at *p* < 0.05 and *p* < 0.001, respectively.

**Table 7:** Variables in the equation (Emotional polarity features).

Variables		B	S.E.	Wald	df	Sig.	EXP(B)	95% C.I. for EXP(B)	
								Lower	Upper
Step 3^a^	Gender	−0.408	0.109	14.012	1	0.000^**^	0.665	0.537	0.867
	Age	0.083	0.034	5.828	1	0.016^*^	0.920	0.860	1.320
	Constant	1.102	0.530	4.372	1	0.038^*^	3.009		

^a^Variable(s) entered on step 3: sex; ^*^ and ^**^ indicate statistical significance at *p* < 0.05 and *p* < 0.001, respectively.

### Binary Logistics Regression Analysis of Demographic Characteristics Affecting Risk Preference

Following Hsee et al. (Hsee and Weber, 1997; Weber, 1999; Wang, 2016) the RP index was defined as follows. In scenario 1, if a participant chose the risky option in Question 1 through Question i − 1 and the sure option in Question i through Question 7, the RP index was defined as i (*i* = 2, 3 ..... 7). In scenario 2, if a participant chose the risky option in Question 7 through Question i and the sure option in Question i-1 through Question 1, the RP index was defined as i (*i* = 2, 3 ..... 7). The RP index equals 1 if a participant chose the sure option in all questions and equals 8 if the participant chose the risky option in all questions. In case the choices of the participant were inconsistent (i.e., chose the sure option in a lower-numbered question but chose the risky option in a higher-numbered question in scenario 1 or chose the sure option in a higher-numbered question and the risky option in a lower-numbered question in scenario 2), the RP index was considered a missing value in further analyses. Consequently, the RP index ranged between 1 and 8, with larger values indicating greater risk-seeking and the midpoint indicating risk neutrality.

According to our research needs, the mean RP index in both scenarios represented the final result, with 4 as the midpoint. Therefore, those participants with an RP index of greater than 4 were categorized as risk-seeking, and the rest were classified as risk avoidance. The risk preference scale part of the questionnaire was then calculated and tallied, and results show that more than half (73.3%) of the participants were risk averse (Table 8).

**Table 8:** Risk preference grouping (*n* = 1,469).

Variables	Group	Frequency (n) or sample percentage (%)
Risk preference	Risk avoidance	1,077 (73.3%)
	Risk-seeking	392 (26.0%)

To further study the population characteristic factors that affect information acceptance, a binary logistic regression analysis of those factors affecting risk preference was conducted. The relationship between the independent and dependent variables was initially examined *via* univariate analyses (i.e., t-test and chi-square test). Results of the univariate analysis are shown in Table 9. The independent sample t-test (in the univariate analysis) showed statistically significant differences in the independent variables of the risk avoidance and seeking groups, including in their gender and grade (*p* < 0.05). Therefore, these two variables were included in the logistic regression model. Results of the omnibus tests of model coefficients (Table 10) show that *p* = 0.000 < 0.05, which indicates that the or value of at least one variable in the fitted model is statistically significant, that is, the model is generally significant.

**Table 9:** Univariate comparison of independent variables between the risk avoidance and risk-seeking groups (*n* = 1,469).

Variables	Risk avoidance group (*n* = 1,077)	Risk-seeking group (*n* = 392)	*x*^2^/*t*	*P*
Gender, male (%)	410(38.1)	225(57.4)	−6.651	0.000**
Grade, grade 10–12 (%)	622(57.8)	263(67.1)	−3.320	0.001*
Place of residence, city (%)	923(85.7)	338(86.2)	−0.256	0.789
Boarding mode, board at school (%)	684(63.5)	243(62.0)	0.531	0.595

* *and* ** denote statistical significance at *p* < 0.05 *and* *p* < 0.001, respectively.

**Table 10:** Omnibus tests of model coefficients.

		Chi-square	df	Sig.
Step 3	Step	11.789	1	0.001
	Block	55.284	2	0.000
	Model	55.284	2	0.000

Among the 392 risk-seeking participants, 225 were male (57.4%) and 263 were senior students (67.1%). Meanwhile, among the 1,077 risk averse participants, 410 were males (38.1%) and 622 were senior students (57.8%). Significant differences were observed in the gender and grade of these two groups [(x)^2^ = − 6.651/−3.320, *p* < 0.01]. Results of the logistic regression model (Table 11) suggest that compared with females, males were more likely to pursue risk when facing risk (or = 2.223, 95% CI: 1.755–2.815). Meanwhile, compared with lower grade students, higher grade students have a higher probability to pursue risk (or = 1.533, 95% CI: 1.198–1.963).

**Table 11:** Variables in the equation.

Variables		B	S.E.	Wald	df	Sig.	EXP(B)	95% C.I. for EXP(B)	
								Lower	Upper
Step3^a^	Gender	0.799	0.121	43.942	1	0.000**	2.223	1.755	2.815
	Grade	0.427	0.126	11.523	1	0.001*	1.533	1.198	1.963
	Constant	−1.659	0.121	187.478	1	0.000**	0.190		

^a^Variable(s) entered on step 3: sex; * *and* ** indicate statistical significance at *p* < 0.05 and *p* < 0.001, respectively.

## Discussion

Florensa showed that mental health education can be used to promote and maintain good mental health (Florensa et al., 2019). The importance of including positive mental health literacy (PMeHL) or knowledge of how to obtain and maintain good mental health as integral components of mental health education among adolescents has also been supported in the literature (Bjornsen et al., 2019). PMeHL, group, and family education all strengthen the content of mental health education programs. However, this study revealed that textual features of information and individual psychological characteristics can also significantly affect the acceptance of mental health education.

### Textual Features of Mental Health Education Materials

Textual features have been proven to influence the acceptance of information (Jiang et al., 2019; Liu et al., 2019), but they are rarely used in the field of mental health education. In this study, teenager students express a strong preference for textual features. Among the four features explored in this work, teenager students preferred digital features, symbols, and positive emotion (*p* < 0.001). Symbols can be used to generate and convey meaning, thus affecting the responses of individuals (Morris, 1966). The prevalence of visual resources on the internet has given rise to visual semiotics, which positively affects the attitudes, intentions, or behavior of users (Valentini et al., 2018; Vanissa et al., 2018). Numbers are commonly used in the health domain as they are often associated with higher scientific credibility (West et al., 2013). Statistical evidence has a stronger influence on beliefs and attitudes than narrative evidence (Zebregs et al., 2014). Previous studies suggest that emotional polarity can affect audience cognition or information feedback (Rao et al., 2020a,b) and that health-related information with positive emotions can be easily received (Gallagher and Updegraff, 2012). These results confirm that textual features also affect information reception in the field of mental health education. Therefore, mental health education programs for teenager students should use positive materials and consider adding the appropriate numbers and symbols in these materials. However, these students did not show any preference for emotional conflict for the time being (*p* > 0.05).

### Influence of Risk Preference on Choice of Textual Features in Mental Health Education Materials

To further consider the persuasive effect of textual features on teenager students, a subgroup analysis was performed to determine whether risk preference affects their choice of textual features. Interestingly, results of the supplementary subgroup analysis suggest that risk preference does not play a regulatory role in the first three text characteristics, thereby verifying that teenager students may express strong preference for numbers, symbols, and positive emotional characteristics. This finding may be ascribed to the adolescents’ constant use of symbols and numbers (Ji et al., 2017), and positive emotions usually have a positive impact on adolescents (Lee et al., 2020). By contrast, among the characteristics of emotional conflict not found preference at present, results of the subgroup analysis show that students who prefer having no emotional conflict in the text showed the characteristics of risk avoidance, or lower grades, or rural or school accommodation (*p* < 0.05). Artyom et al. confirmed that emotions strongly influence cognitive and emotional conflict processing and highlighted the complexity and heterogeneity of the interaction of emotion with different types of conflicts (Zinchenko et al., 2015). Teenagers who lack family warmth may be more emotional (Ching and Wu, 2018). We also found that the probability of men choosing texts with positive emotional polarity is 33.5% lower than that of women; Moreover, the probability of choosing positive emotional polarity reduced by 8% for every 1-year-old increase in age as well. The difference between gender and age in reading has long been confirmed (Zhu et al., 2018).

### Demographic Characteristics and Mental Health Education Materials

Meanwhile, findings of this work suggest that among the 1,469 participants, only 392 showed risk-seeking behaviors when faced with risk. Lauriola found that compared with other groups, the adolescent was more reluctant to take risks (Lauriola and Levin, 2001). Risk avoidance people prefer no emotional conflict. Therefore, for the majority of the teenager students, the stimulation materials should not introduce too strong emotional conflict.

The findings of this work also confirm that compared with females (lower grade students), males (higher grade students) are more inclined to risk-seeking as confirmed by the differences in their race, grade level, parent education level, educational aspirations, and frequency of risk behavior (Shukla et al., 2016). Specifically, females reported lower levels of mental wellbeing, perceived health, and life satisfaction compared with males (Organization, W.H, 2016). As for the risk decision-making performance of junior middle school students, males choose more adverse options with higher losses, whereas females choose more favorable options with lower losses (Xiaoxiao et al., 2018).

### Limitations

We realize that there may still be some problems in our research, such as the balance of urban and rural distribution of the investigated population. We have tried to balance the urban and rural distribution of the surveyed students, but in fact, the four schools we surveyed are distributed in different regions of Chongqing, China. The students of these schools also come from different places, so the final result will be random. At the same time, due to the epidemic of COVID−19 and regional policies, we were unable to carry out more population surveys. Moreover, in order to ensure the authenticity and accuracy of the questionnaire, we did not conduct online survey. In the next stage our team will carry out more follow-up investigations of rural middle schools, of course.

## Conclusion

Textual features influence the persuasive effect of mental health education information for teenager students. The effect of mental health education and promotion strategies should be maximized by adding textual features, such as numbers, symbols, and positive emotions, in mental health education materials. At the same time, the regulatory effect of risk preference is only reflected in emotional conflict. Students who prefer having no emotional conflict in the text showed the characteristics of risk avoidance, or lower grades, or rural or school accommodation. Gender and age also play a regulatory role in the characteristics of emotional polarity. Female and lower grades prefer texts with positive emotions. Moreover, most teenager students are risk avoidance. Therefore, the stimulation materials for these students should not introduce too much emotional conflict. Female and lower grade students also have a higher inclination to avoid risks compared with their male and higher grade counterparts. Therefore, educational materials with different text characteristics should be used for teenager students with different characteristics to generate better educational effects. For example, for girls in lower grades, lovely emoticons can be added to the text materials, and the emotion should be positive without strong emotional conflict.

## Data Availability Statement

The raw data supporting the conclusions of this article will be made available by the authors, without undue reservation.

## Ethics Statement

The studies involving human participants were reviewed and approved by the Ethics Committee of the Chongqing Medical University (record number 2018011). The participants gave their written informed consent before answering the questionnaire.

## Author Contributions

All authors significantly contributed to the work reported whether in the study design, execution, data collection, data analysis, and data interpretation. XH, MJ, and ZZ designed the experiments. XH, MJ, LK, ZZ, JL, and SW collected data. MJ wrote the paper and analyzed the data. YL and XZ helped analyse the data. Each author took part in drafting, revising, or critically reviewing the article and gave his/her final approval of the version to be published.

## Funding

This study is funded by the Project of Humanities and Social Sciences of the Ministry of Education of China (Grant Number 20YJCZH043), the Intelligent Health Knowledge Discovery and Information Service of the Chongqing Medical University (Grant Number ZHYX2019006), the Intelligent Medical Foundation of the Chongqing Medical University (Grant Number YJSZHYX202123), and the Chongqing Graduate Scientific Research Innovation Project of the Chongqing Municipal Education Commission (Grant Number CYS20225), Chongqing Medical University Future Medical Innovation Team Support Program (W0054).

## Conflict of Interest

The authors declare that the research was conducted in the absence of any commercial or financial relationships that could be construed as a potential conflict of interest.

## Publisher’s Note

All claims expressed in this article are solely those of the authors and do not necessarily represent those of their affiliated organizations, or those of the publisher, the editors and the reviewers. Any product that may be evaluated in this article, or claim that may be made by its manufacturer, is not guaranteed or endorsed by the publisher.
