# Supplementary material for: Textual Features and Risk Preference Effects on Mental Health Education Among Teenager Students in Chongqing, China
Source: Front Psychol. 2022 May 26;13:911955. doi: 10.3389/fpsyg.2022.911955 (PMC9181320; doi:10.3389/fpsyg.2022.911955)
Supplement: Supplementary file 1 [file Data_Sheet_1.docx]

**A survey of teenager students' preference for textual features**

Hello, dear students. Welcome to our survey. Please answer the following questions according to your actual situation and experience. There is no standard answer. The real answer of each student is helpful to our research. This questionnaire is only for academic research. We will keep your information confidential! Thank You for Your Cooperation!

Informed consent signature：

Research Group on health information frame effect of Chongqing Medical University

Contact person: Mengyao Jiang, Xiaorong Hou Tel.: 17725029425

1. **Demographic information survey**

1. Gender: A. Male B. Female（ ）

2. Age:（ )

3.Where is your place of residence？ A. Urban B. Rural（ ）

4. What is your type of school? A. Boarding at school B. Day school（ ）

1. **Please answer the following parts according to your own real thoughts**

(Here are four scenarios about mental health education for teenager students. There is no standard answer, but you will find that their expressions are different after careful reading. Please circle the numbers after each headline according to your preferences.)

5 = totally like, 4 = like , 3 = neutral, 2 = dislike, and 1 = totally dislike **Examples：**( 5 4 3 2 1 )

**1、During the epidemic period, all schools carried out online teaching, which not only brought convenience, but also easy to internet addiction. In order to reduce everyone's dependence on the Internet, the school will hold relevant theme lectures. Please score the following two lecture headlines according to your preferences.**

- From "Social phobia" to "Social Whiz" -- a step-by-step guide to social nerds ( 5 4 3 2 1 )
- Correctly deal with the change of social interaction and learn how to become a social talent ( 5 4 3 2 1 )

**2、During the epidemic, due to the reduction of social communication, it is easy to cause anxiety. In order to let everyone master some methods to resolve anxiety, the head teacher mix these methods into a short video of health education. Please score the following two short video headlines according to your preferences.**

- 3 tips help you to say goodbye to all anxiety ( 5 4 3 2 1 )
- Help you to say goodbye to all anxiety ( 5 4 3 2 1 )

**3、During the epidemic, depression and anxiety increased among adolescents. The propaganda department of the school wrote a popular science article about emotion controlling. Please rate the headlines of the following two articles according to your preferences.**

- Anxiety, panic, teach you how to prevent the epidemic, refuse to panic ( 5 4 3 2 1 )
- Anxiety (• _ •)? Panic! ? Teach you how to prevent the epidemic, refuse to panic!! ( 5 4 3 2 1 )

**4、During the epidemic, there was no place to vent emotions, leading to psychological pressure. Therefore, the school will produce a health education video about how to release psychological stress. Please score the following two video headlines according to your preferences.**

- Isolated, the world becomes anxious ( 5 4 3 2 1 )
- Being with us, the world is bright ( 5 4 3 2 1 )

**三、The following are scenario assumptions. Please answer them according to your real thoughts**

| **Suppose you won the lottery last week, now you are contacted about the prize. You can choose between A and B. please judge according to your real thoughts.**  **^a^H means the coin head shows up**  **^b^T means the coin tail shows up** | | | | |
| --- | --- | --- | --- | --- |
| Set | Question | A | B | Answer |
| Set Ⅰ | 1 | receive $400 for sure | flip a coin; receive $2000 if H^a^ or $0 if T^b^ |  |
|  | 2 | receive $600 for sure |  |  |
|  | 3 | receive $800 for sure |  |  |
|  | 4 | receive $1000 for sure |  |  |
|  | 5 | receive $1200 for sure |  |  |
|  | 6 | receive $1400 for sure |  |  |
|  | 7 | receive $1600 for sure |  |  |
|  | | | | |
| Set Ⅱ | 1 | receive $20 for sure | flip a coin; receive $100 if H^a^ or $0 if T^b^ |  |
|  | 2 | receive $30 for sure |  |  |
|  | 3 | receive $40 for sure |  |  |
|  | 4 | receive $50 for sure |  |  |
|  | 5 | receive $60 for sure |  |  |
|  | 6 | receive $70 for sure |  |  |
|  | 7 | receive $80 for sure |  |  |

| **Suppose someone was injured last week because of your traffic violation, you need to compensate the injured. You can choose between A and B. please judge according to your real thoughts.** | | | | |
| --- | --- | --- | --- | --- |
| Set | Question | A | B | Answer |
| Set Ⅰ | 1 | pay $400 for sure | flip a coin; pay $2000 if H^a^ or $0 if T^b^ |  |
|  | 2 | pay $600 for sure |  |  |
|  | 3 | pay $800 for sure |  |  |
|  | 4 | pay $1000 for sure |  |  |
|  | 5 | pay $1200 for sure |  |  |
|  | 6 | pay $1400 for sure |  |  |
|  | 7 | pay $1600 for sure |  |  |
|  | | | | |
| Set Ⅱ | 1 | pay $20 for sure | flip a coin; pay $100 if H^a^ or $0 if T^b^ |  |
|  | 2 | pay $30 for sure |  |  |
|  | 3 | pay $40 for sure |  |  |
|  | 4 | pay $50 for sure |  |  |
|  | 5 | pay $60 for sure |  |  |
|  | 6 | pay $70 for sure |  |  |
|  | 7 | pay $80 for sure |  |  |
